# Bibliometric trend analysis of non-conventional (alternative) therapies in veterinary research

**DOI:** 10.1080/01652176.2022.2142318

**Published:** 2022-11-11

**Authors:** Karolina Domingues, Nuno Henrique Franco, Isilda Rodrigues, George Stilwell, Manuel Magalhães-Sant’Ana

**Affiliations:** a Instituto de Investigação e Inovação em Saúde, Universidade do Porto, Porto, Portugal; b Departamento de Educação e Psicologia, Universidade de Trás-os-Montes e Alto Douro, Vila Real, Portugal; c Centro de Investigação e Intervenção Educativas, Faculdade de Psicologia e de Ciências da Educação, Universidade do Porto, Porto, Portugal; d Centro de Investigação Interdisciplinar em Sanidade Animal, Faculdade de Medicina Veterinária, Universidade de Lisboa, Lisboa, Portugal; e Laboratório Associado para Ciência Animal e Veterinária, Lisbon, Portugal

**Keywords:** literature search, text mining, evidence-based medicine, complementary therapies, alternative therapies, acupuncture, phytotherapy, homeopathy, essential oils, veterinary medicine

## Abstract

**Background:** There is an increased interest in Non-Conventional Therapies (NCTs), often referred to as complementary and alternative medicines, in veterinary clinical practice.

**Aim:** To map the bibliometric outputs of NCTs in veterinary medicine, and identify which are most prevalent, and the extent to which their publishing has increased.

**Methods:** Text mining algorithms were applied to detect 17 NCTs-related terms (*acupuncture*, *ayurveda/ayurvedic*, *traditional Chinese medicine*, *traditional medicine, chiropractic*, *electroacupuncture*, *essential oil*, *plant extract, ethnopharmacology*, *herbal medicine*, *homeopathy*, *low-level laser therapy*, *medicinal plant, natural product*, *osteopathy*, *phytotherapy*, and *massage*) in the title, abstract or keywords of all retrievable literature until 2020 under the PubMed MeSH term ‘veterinary’ (*N* = 377 556). Point prevalence, incidence by decade and cumulative incidence were calculated.

**Results:** Bibliometric trend analysis revealed an overall increase in NCTs-related terms over the last 20 years, with a substantial growth of studies mentioning *plant extracts*, *essential oils* and *medicinal plants. Traditional Chinese medicine*, *herbal medicine* and *natural product* have also increased in the same period, although their numbers remain low. Conversely, reference to *acupuncture* has decreased in the last decade when compared with the previous decade, whereas references to *homeopathy*, *electroacupuncture*, *osteopathy* and *chiropractic* remained scarce, suggesting that their use in veterinary clinical practice may not be based on published evidence.

**Conclusion:** Further reviews to explore this issue are warranted, differentiating secondary from primary literature, and assessing relevance and methodological quality of individual studies, following the principles of evidence-based veterinary medicine.

## 1. Introduction

Evidence-based medicine (EBM) consists of making the best possible decision for individual clinical cases, by grounding them on the best available scientific evidence (Sackett et al. 1996). Likewise, information and evidence-based approaches are recognized as important professional skills for graduate veterinarians (Cake et al. 2016), while evidence-based veterinary medicine (EBVM) has been established as a research field of its own. EBVM is arguably more challenging to accomplish than EBM, due to the dearth of robust, high-quality evidence in veterinary medicine (Mills 2015). Hence, veterinary practitioners often rely upon their practical experience or on empirical evidence, at least as much as in their skills to analyse and interpret data from published studies (Holmes 2009; Turner and Royle 2015), making them particularly vulnerable to heuristic reasoning and cognitive biases (McKenzie 2014; Lees et al., 2017).

Parallel to the rise of EBVM, there has been an alleged increase in popularity of non-conventional therapies (NCTs) in veterinary practice, often referred to as complementary and alternative veterinary medicines (Gilberg et al. 2021; Keller et al. 2021; Stanossek and Wehrend 2022), “a diverse group of practices and products not considered part of conventional (main-stream) medicine” (British Small Animal Veterinary Association 2022). Although there is no universal definition for NCTs, in this paper the term will be used to denote therapies based on non-scientific principles and/or lacking reliable scientific evidence of effectiveness (McKenzie 2012), which include traditional Chinese medicine, acupuncture, homeopathy, osteopathy, chiropractic and naturopathy, to name but a few (Zollman and Vickers 1999).

The use of NCTs in veterinary practice is problematic for several reasons. There is currently lack of reliable evidence that these therapies can benefit animal patients (Bergh et al. 2021). In human medicine, the use of alternative therapies has been associated with the delaying of conventional therapies (Davis et al. 2006; Akhtar et al. 2018) and an increased risk of death from curable cancer (Johnson et al. 2018). There is often an overlap between NCTs and anti-scientific movements, such as “big pharma” conspiracies or vaccine hesitancy (Gaudino and Robison 2012; Attwell et al. 2018), a concern recently raised in veterinary medicine (Loeb 2019; Whitehead et al. 2019). At a time where the fight against antimicrobial resistance is at its peak, the interest in NCTs as an alternative to the use of antimicrobials has increased, but good-quality evidence is still lacking (Baars et al. 2019).

To this date, no attempt has been made to map the bibliometric outputs of NCTs in veterinary medicine, and namely in identifying which are most prevalent, and the extent to which their publishing has increased. To inform this debate, we applied text mining to examine publication trends, namely prevalence and incidence, of NCT-related keywords in the veterinary research literature.

## 2. Material and methods

To determine which NCTs are present in the published veterinary literature, we first performed a preliminary search on three academic databases (PUBMED, Scopus and Web of Science Core Collection) using the advanced search query and the terms *(("alternative therapies" OR "complementary therapies") AND (veterinary))*. We then applied filters to exclude reviews, books and guidelines, as well as *“other animals”*. For each database, the results were exported to .RIS format and imported into VOS-Viewer for the assessment of keyword co-occurrence and word cloud visualization. The minimum occurrence for keyword was set as *n* = 2. Terms were checked for applicability and relevance. This screening process was performed by KD and discussed among all authors until a consensus was reached. The terms “physical therapy”, “electrostimulation”, “cryotherapy” and “hydrotherapy” were discarded because these practices rely mostly on established (conventional) physiotherapic techniques (Millis and Levine 2014) that fall outside the definition of NCTs used in this research. The term “osteopathy” did not emerge from this initial search but was nonetheless included, due to strong evidence that veterinary osteopathy is being researched (Thelwall 2021) and practiced (Pusey et al. 2010). A final list of 17 NCT-related key terms was obtained: *acupuncture*, *ayurveda/ayurvedic*, *traditional Chinese medicine*, *traditional medicine, chiropractic*, *electroacupuncture*, *essential oil*, *plant extract, ethnopharmacology*, *herbal medicine*, *homeopathy*, *low-level laser therapy*, *medicinal plant, natural product*, *osteopathy*, *phytotherapy*, and *massage*.

**Figure 1.**
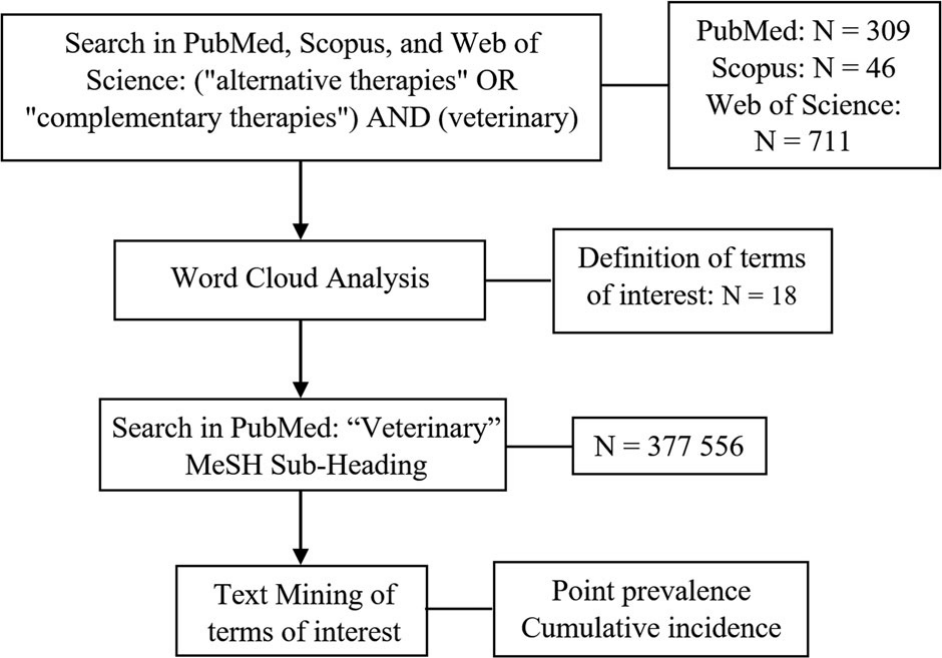
Flowchart of the sequence of procedures used to analyse the trends of Non-Conventional Therapies in the veterinary literature.

After the screening process for word cloud analysis and the selection of the terms of interest, a new search was performed in the PubMed database for items published until 2020 (no lower limit for year of publication), using the “Veterinary” Medical Subject Headings (MeSH) Sub-Heading. For this analysis we used only the PubMed database since the MeSH taxonomy of index terms allows for an objective and targeted search. The results of this search (*N* = 377 556) were exported to .RIS format, turned into .XML using the ENDNOTE reference manager, and then analysed using Python programming language and text mining packages (code available as supplementary material at https://osf.io/fcrgm/?view_only=e6063ddf2e9440a78782816b1f34499e) to find the previously defined 17 NCT-related terms in the title, keywords and/or abstract of each reference. The hits were checked by MMS to ensure that the terms were being used within the context of NCTs, and validated by remaining co-authors. For example, “massage” is used in contexts other than physical rehabilitation (e.g. uterine or cardiac massage). The term osteopathy is also used to describe a bone disease (e.g. craniomandibular osteopathy in the dog or hypertrophic osteopathy in cats). The first derivative and non-linear fit were calculated using GraphPad Prism Software, and the graphs were plotted using the same software. A flowchart summarizing the main research steps can be found in Figure 1.

## 3. Results

The preliminary search for complementary and alternative veterinary therapies yielded 309 results in Pubmed, 46 in Scopus and 711 in Web of Science. The word cloud results, as well as the relevant terms filtered, are available as supplementary material in OSF platform (https://osf.io/xc48r/?view_only=e6063ddf2e9440a78782816b1f34499e).

**Figure 2.**
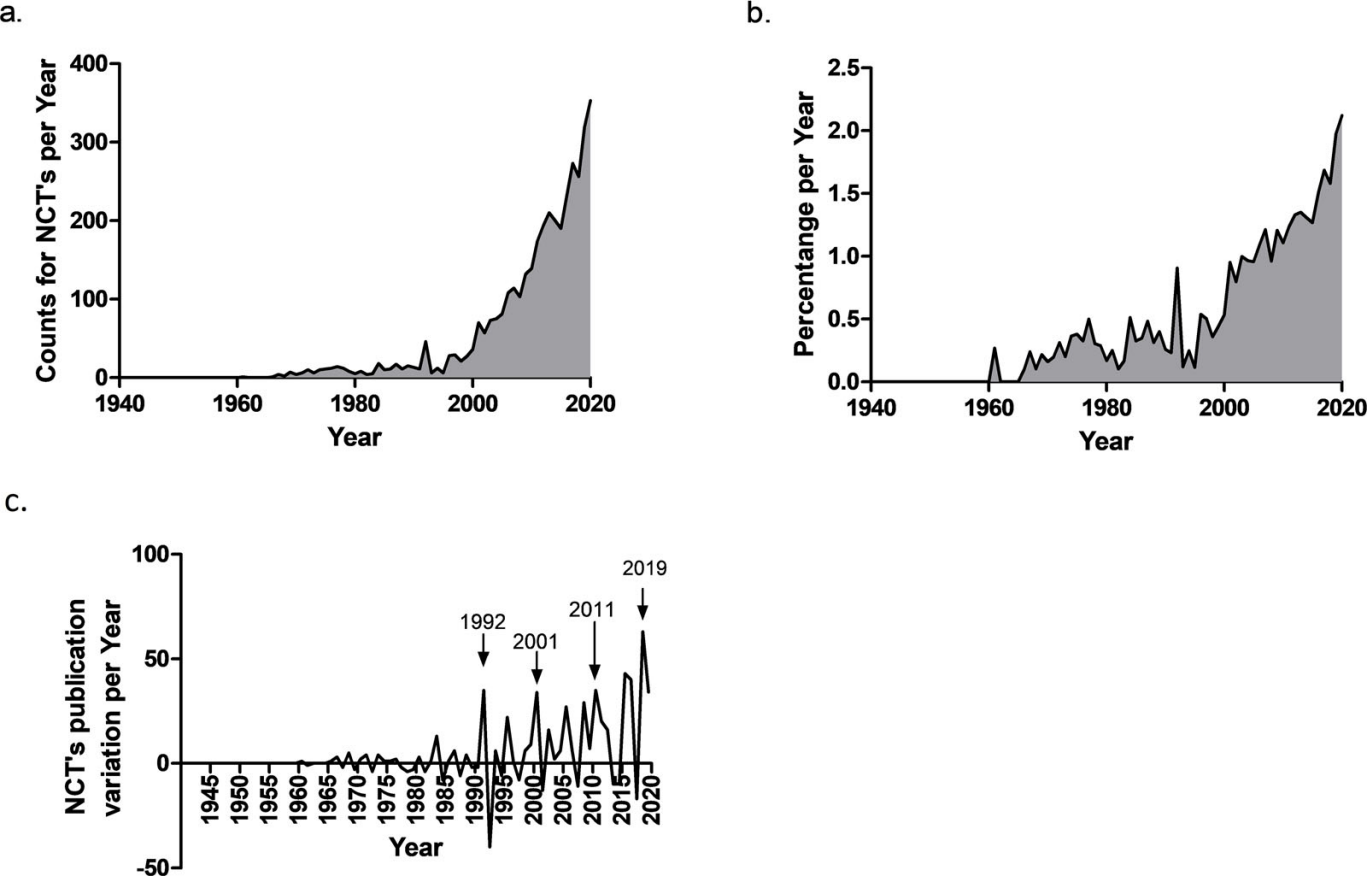
Number of NCTs-related keyword counts per year (cumulative incidence) (a), percentage of publications with NCTs-related keywords per year (point prevalence) (b) and the first derivative showing the inflexion points (c).

From all the references in PubMed under the “Veterinary” MeSH Subheading (*N* = 377 556), we observed 3061 hits stating NCTs in either the title, abstract, or keywords (full results available in OSF platform (https://osf.io/cjv7a/?view_only=e6063ddf2e9440a78782816b1f34499e)). The overlap between key terms (e.g. *acupuncture* and *traditional Chinese medicine* often coincided in the same article) precludes calculating the total percentage of references that mention each NCT. The text mining analysis indicated that the first veterinary literature using NCT-related keywords dates back to 1961, with a paper reporting the experimental use of oncolysin to treat canine mammary tumours (keyword: plant extract) (Table 1). No NCT-related keywords were found for the following four years and counts per year remained low (between 1 and 18) until 1991. The first reference to homeopathy is from 1971 and acupuncture is first mentioned in a brief letter to the editor of the Journal of the American Medical Association, in 1972 (Table 1), although the first empirical study is arguably a 1975 case report of a reportedly successful acupuncture treatment of cervical disc disease in a dog (Buchli 1975). In 1992 there was a surge in NCT-related keywords (42 hits, Figure 2c), mostly driven by research in acupuncture (*N* = 23). Within the next 8 years, mentions to NCTs ranged between 5 and 35, with a marked increase observable in the last two decades (Figure 2a). The term osteopathy is used for the first time in the context of NCT in 2010 (Table 1) and only two other hits were identified. Publications mentioning at least one NCT-related keyword represent roughly 2.1% of all academic references indexed in PubMed in 2020 under the ‘veterinary’ MeSH term (Figure 2b). By calculating the first derivative, several publication peaks were identified, namely 1992, 2001, 2011 and 2019 (Figure 2c).

**Table 1.** First mention to thirteen terms related to Non-Conventional Therapies in the title, abstract or keyword from all retrievable literature, published until 2020, under the PubMed MeSH term ‘veterinary’ (*N* = 377 556).

Term	Year	First author	Title	MeSH terms	Journal
Plant extract	1961	Pelner, L	Host-tumor antagonism. 18. Experimental treatment of breast cancer in dogs with an hydrolysate of certain plant seeds (oncolysin)	Animals, Antineoplastic Agents/therapy*, Breast Neoplasms/veterinary*, Dogs, Humans, Neoplasms*, Plant Extracts*	Journal of the American Geriatrics Society
Medicinal plant	1967	Sharma, LD	Anthelmintic screening of three indigenous medicinal plants against Axcaridia galli in poultry	Animals, Anthelmintics*, Nematode Infections/drug therapy/veterinary*, Plants, Medicinal*, Poultry Diseases/drug therapy*	Indian Veterinary Journal
Phytotherapy	1967	Mullenax, CH	Clinical observations of an effective ruminatoric	Animals, Cattle, Cattle Diseases/drug therapy* Phytotherapy*, Plants, Medicinal*, Plants, Toxic*, Rumen/drug effects*, Stomach Diseases/drug therapy/veterinary, Veratrum/therapeutic use*	Veterinary Medicine, Small Animal Clinician
Homeopathy	1971	Bordet, R	[Dental pathology in horses and dogs]	Animals, Dog Diseases*, Dogs, Homeopathy, Horse Diseases*, Horses, Tooth Diseases/pathology/prevention & control/therapy/veterinary*	Revue Francaise d‘Odonto-stomatologie
Acupuncture	1972	Satory, JJ	Acupuncture for horses	Acupuncture Therapy/veterinary*, Animals, Horses*	Journal of the American Medical Association
Massage	1975	Downer, AH	Physical therapy in the management of long bone fractures in small animals.	Animals, Casts, Surgical, Dog Diseases/therapy, Dogs, Exercise Therapy/veterinary, Femoral Fractures/therapy/veterinary, Fracture Fixation, Internal/veterinary, Fractures, Bone/therapy/veterinary*, Hot Temperature, Humeral Fractures/therapy/veterinary, Hydrotherapy/veterinary, Immobilization, Massage/veterinary, Physical Therapy Modalities/veterinary*, Radius Fractures/therapy/veterinary, Rest, Swimming, Ulna Fractures/therapy/veterinary, Ultrasonic Therapy/veterinary	The Veterinary Clinics of North America
Essential oil	1977	Wilson, BJ	Perilla ketone: a potent lung toxin from the mint plant, Perilla frutescens Britton	Animals, Cattle, Cattle Diseases/chemically induced, Furans/isolation & purification/toxicity*, Lethal Dose 50, Lung/drug effects*, Mice, Monoterpenes, Plants, Toxic/analysis*, Pulmonary Edema/chemically induced, Pulmonary Emphysema/veterinary, Rats, Sheep, Terpenes/isolation & purification/toxicity*, Toxins, Biological/*isolation & purification	Science
Ayurveda/ayurvedic	1978	Dange, BN	Clinical trials with Myron and Aloes Compound in cases of bovine infertility	Aloe*, Animals, Buffaloes*, Cattle, Cattle Diseases/drug therapy*, Drug Combinations, Female, Infertility, Female/drug therapy/veterinary*, Medicine, Ayurvedic*, Phytotherapy, Plant Extracts, Plants, Medicinal Pregnancy	Indian Veterinary Journal
Electroacupuncture	1980	Cheng, R	Electroacupuncture elevates blood cortisol levels in naive horses; sham treatment has no effect	Acupuncture Therapy/methods*, Adrenal Cortex/metabolism, Adrenocorticotropic Hormone/metabolism, Animals, Arthritis/therapy/veterinary, Electric Stimulation Therapy/methods*, Endorphins/metabolism, Horse Diseases/therapy, Horses/metabolism*, Hydrocortisone/blood*, Pain/veterinary, Pain Management, Pituitary Gland, Anterior/metabolism, Time Factors	International Journal of Neuroscience
Chiropractic	1984	Jagger, DH	Alternative veterinary medicine	Acupuncture Therapy/veterinary, Animals, Chiropractic/veterinary, *Education, Veterinary, Homeopathy/veterinary	The Veterinary Record
Traditional chinese medicine	1992	Limehouse, JB	Oriental concepts of acupuncture	Acupuncture Therapy/veterinary*, Animals, Blood Physiological Phenomena, Humans, Medicine, Chinese Traditional*, Meridians, Veterinary Medicine/methods*, Yin-Yang	Problems in Veterinary Medicine
Low-level laser therapy	1996	Ghamsari, SM	Histopathological effect of low-level laser therapy on sutured wounds of the teat in dairy cattle	Animals, Cattle/injuries*/physiology/surgery, Cattle Diseases/physiopathology/radiotherapy, Female, Laser Therapy*, Mammary Glands, Animal/injurie*s/radiation effects/surgery, Suture Techniques/veterinary*, Wound Healing/physiology/radiation effects*, Wounds and Injuries/pathology/radiotherapy/veterinary	Veterinary Quarterly
Osteopathy	2010	Haussler, KK	The role of manual therapies in equine pain management	Analgesia/veterinary, Animals, Horse Diseases/therapy*, Horses, Musculoskeletal Manipulations/veterinary*, Pain/veterinary*, Pain Management, Physical Therapy Modalities/*veterinary	Veterinary Clinics of North America -Equine Practice

**Figure 3.**
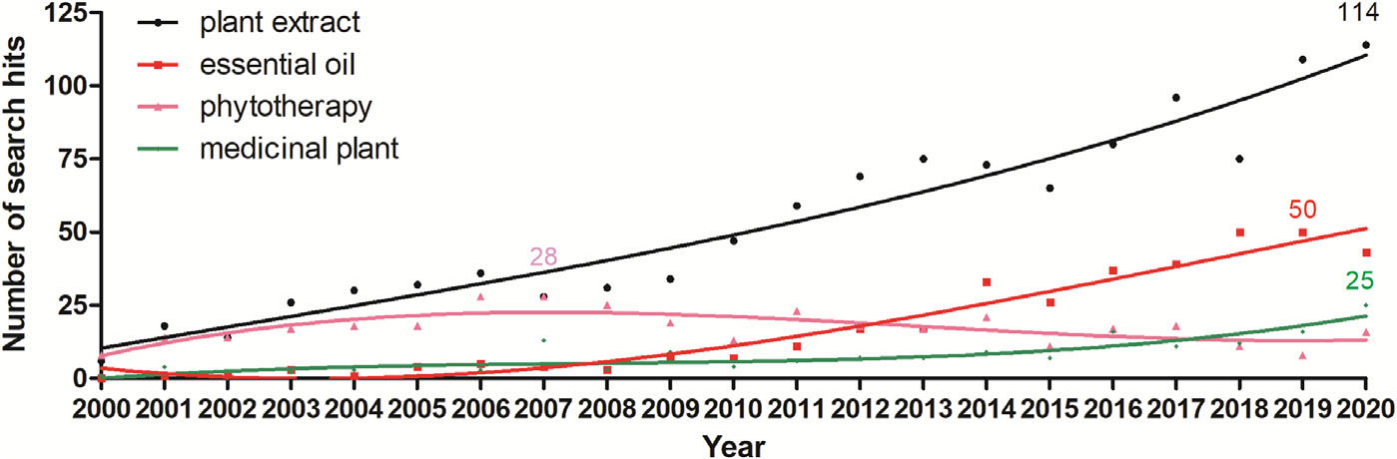
Mapping publications per year (since the year 2000) according to highly occurring keywords (more than 10 hits per year, on average, in the last decade): *plant extract*, *essential oil*, *phytotherapy* and *medicinal plant*. The curves were fitted using a second order polynomial nonlinear fit (cubic).

**Figure 4.**
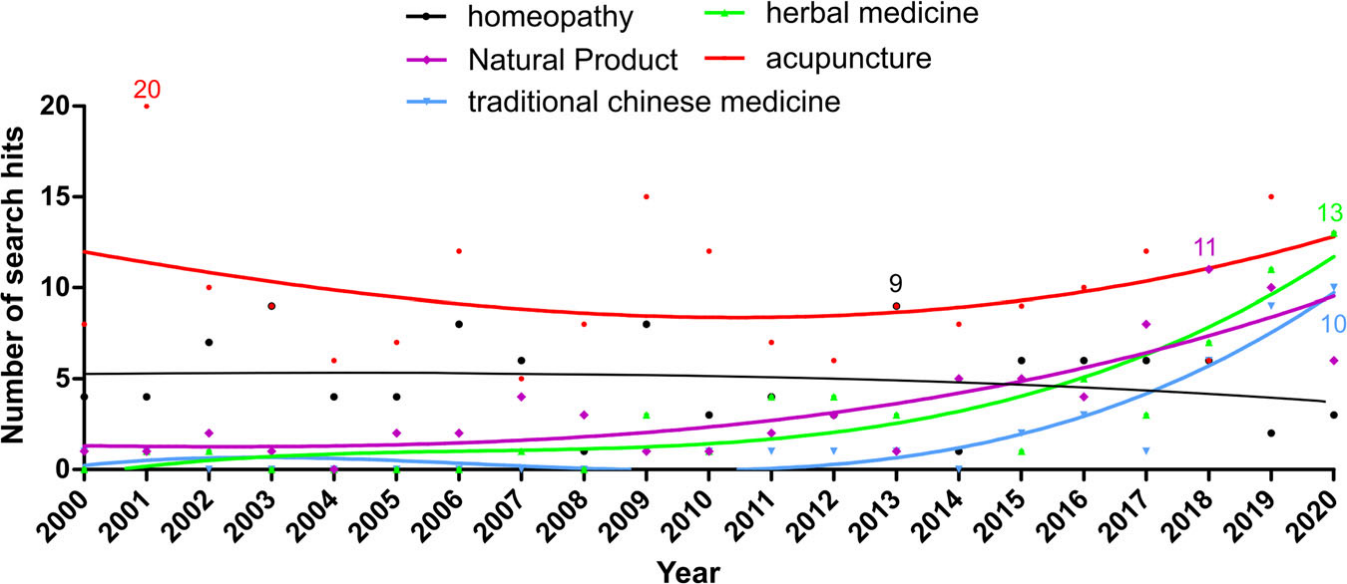
Mapping publications per year (since the year 2000) referring to less-occurring NCTs-related keywords (less than 10 hits per year, on average, in the last decade): *acupuncture*, *herbal medicine*, *natural product*, *traditional Chinese Medicine* and *homeopathy*. The curves were fitted using a second order polynomial nonlinear fit (cubic).

When considering the last two decades, we found a marked difference between two groups of keywords, in terms of their occurrence in the literature (Figures 3 and 4). The most prevalent term is *plant extract* (Figure 3, black line), which has increased substantially (Table 2). This was observable also, yet mostly within the last decade, for studies mentioning *essential oils* (Figure 3, red line) and *medicinal plant* (Figure 3, green line), whereas studies mentioning *phytotherapy* have fluctuated little over the last 20 years (Figure 3 pink line), with a 17.5% decrease in the last decade when compared with the previous decade (Table 2).

**Table 2.** Number of hits of terms related to Non-Conventional Therapies per decade and the percentage difference from the previous decade (in brackets). Terms are organised in descending order of incidence in the last decade (2011–2020).

Term / Time period	1971–1980	1981–1990	1991–2000	2001–2010	2011–2020
Plant extract	37	26 (-29.7%)	64 (146.2%)	296 (362.5%)	815 (175.3%)
Essential oil	2	1 (-50%)	1 (0)	37 (3600%)	323 (773%)
Phytotherapy	5	1 (-80%)	18 (1700%)	194 (977.8%)	160 (-17.5%)
Medicinal Plant	6	2	5 (+150%)	42 (740%)	116 (176.2%)
Acupuncture	30	45 (50%)	59 (31.1%)	104 (76.3%)	95 (-8.7%)
Herbal medicine	0	2	5 (150%)	7 (40%)	56 (700%)
Natural product	0	0	2	17 (750%)	55 (223,5%)
Homeopathy	1	12 (1100%)	24 (100%)	54 (125%)	46 (-14.6%)
Traditional Chinese medicine	0	0	7	3 (-57.1%)	34 (1033.3%)
Traditional medicine	0	1	3 (200%)	9 (200%)	33 (266,7)
Ethnopharmacology	0	0	0	7	21 (200%)
Electroacupuncture	1	5 (400%)	4 (20%)	30 (650%)	20 (-33.3%)
Low-level laser therapy	0	0	1	3 (200%)	16 (433.3%)
Chiropractic	1	1 (0)	9 (800%)	10 (11.1%)	14 (40%)
Massage	2	0	7	12 (71.4%)	14 (16.7%)
Ayurveda/ayurvedic	1	0	0	4	4 (0)
Osteopathy	0	0	0	1	2 (100%)

Another group of keywords appear less frequently, with fifteen or fewer hits per year (except for an outlier: 20 hits for acupuncture in 2001). Among these, *acupuncture* is the most prevalent but has now plateaued (8.7% decrease within the last decade), whereas *herbal medicine*, *natural product, traditional Chinese medicine*, *traditional medicine*, and *ethnopharmacology* have increased substantially in the same period, although their numbers remain low (Table 2). *Homeopathy* and *electro-acupuncture* have decreased in the last decade (-33.3% and −14.6% respectively) (Figure 4). The remaining NCTs-related keywords, including *low-level laser therapy, chiropractic, massage, ayurveda/ayurvedic* and *osteopathy*, shows only a few yearly hits. Most of these did not generate enough hits for fitting into a curve and are not represented.

## 4. Discussion

This preliminary bibliometric study aimed to quantify the publication trends of Non-conventional Therapies (NCT) in the veterinary literature. We measured point prevalence, incidence by decade and cumulative incidence of NCT-related keywords, *via* a text mining approach. To the authors’ knowledge, this study provides the first mapping and temporal trend analysis of NCTs in veterinary research.

Since the first hit in 1961, scientific references mentioning NCTs fluctuated little for almost forty years, with a marked increase since the beginning of this century. Results partially confirm previous claims that the interest in NCTs in veterinary practice has been increasing, especially within the last few decades (Magalhães-Sant’Ana 2019; Gilberg et al. 2021; Keller et al. 2021), yet mostly in regard to phytotherapy, as the largest increase in papers containing NCT-related terms in the veterinary literature in the last decade refer to use of medicinal plants, herbal extracts and essential oils. Veterinary research based on phytotherapy involves the use of traditional formulations of medicinal plants as alternatives to commercial drugs, including antimicrobials (Grützner 2019), and is not, in principle, un-scientific. Indeed, finding new active ingredients is at the heart of the development of new treatment modalities and some of these may arrive from medicinal plants. However, lack of quality control measures to identify the pharmacological composition of these formulations, coupled with the lack of well-designed clinical trials, remain a serious concern (Coghlan et al. 2012). Our results also suggest that the rising trend of plant-based research may also be related with an increased interest in traditional medicine, and traditional Chinese medicine in particular, a matter of One Health concern, especially after the COVID-19 pandemic (Sturgeon 2020). In effect, mention to traditional Chinese medicine has risen more than 1000% in the veterinary literature in the last decade (2011–2020) when compared to the previous decade (Table 2).

It is noteworthy that research studies mentioning acupuncture, electroacupuncture, chiropractic and homeopathy are relatively scarce, and have remained so in the last few decades, whereas research on animal osteopathy is virtually inexistent. This can reflect an increasing interest in NCTs that apply (at least some) active ingredients (e.g. essential oils) at the detriment of placebo-based practices such as acupuncture (Colquhoun and Novella 2013; Magalhães-Sant’Ana 2019) or homeopathy (Lees et al., 2017). This also seems to contradict the claim made by Thelwall that there is substantial veterinary research interest in acupuncture, chiropractic, homeopathy, and osteopathy (Thelwall 2021). The results also suggest that the reputation of these (arguably popular) veterinary NCTs is not sustained by published empirical evidence. Likewise, a recent systematic review of 24 NCTs used in animals (dogs, cats and horses) failed to find eligible evidence for 15 of those therapies and, when the evidence existed, it was deemed to have between a moderate and a high risk of bias (Bergh et al. 2021). Altogether, these findings should make us reflect on whether we should accept as good practice therapies that are not based on a robust body of knowledge.

Three reasons have been suggested for justifying the dearth of evidence in veterinary NCTs: a) lack of funding for NCT research, especially for those therapies not supported by pharmaceutical companies; b) inaccessibility to research which is published in languages other than English and c) a publication bias against NCTs (Memon and Sprunger 2011; Fan 2015). However, research funding is increasingly based upon the scientific merits of individual projects, especially those funded by public agencies, and there is no evidence that a publication bias against NCTs exist. Regarding language, most published research provides at least some bibliographic information in English that enables their inclusion in scientific databases. In effect, our research retrieved several studies in foreign languages (cf. Table 1) where at least the English title, abstract or keywords were available. A recent systematic review in human medicine found negligible impact of restricting systematic reviews to English-language publications in conventional medicine but reported changes in treatment effects and statistical significance in the case of alternative medicines, probably due to overestimation of treatment effects and higher risk of bias in non-English publications (Dobrescu et al. 2021).

A few limitations of this study need to be acknowledged. The rise in published NCT studies in the last 20 years can be partially due to an increase in NCT journals, a phenomenon heightened by the Open Access revolution in the last decade (Fan 2015; Ng 2021). However, assessing the type and quality of the journals in which the papers found were published was beyond the aims of this preliminary investigation. Since our database for this trend analysis comprises all scientific literature published under the "Veterinary" MeSH sub-heading, it includes basic research, clinical studies, and even reviews, comments and letters to the editor. Thus, this may lead to an overestimation of the actual number of original publications providing evidence of therapeutic NCTs. It should hence be stressed that original research results grounding the use of NTCs in clinical practice may be even scarcer in the overall veterinary literature than these preliminary results suggest.

In order to address the problem of overestimation of published NCTs research and to refine our study, further and more detailed bibliographic investigation is needed to assess the clinical relevance of the existing literature, as well as the quality and reliability of each individual study. To that end, our group is currently on the final stages of preparing a systematic review protocol to assess these matters.

## Supplementary Material

Supplemental Material
