# Supplementary material for: Bibliometric trend analysis of non-conventional (alternative) therapies in veterinary research
Source: Vet Q. 2022 Nov 11;42(1):192–8. doi: 10.1080/01652176.2022.2142318 (PMC9662055; doi:10.1080/01652176.2022.2142318)
Supplement: Supplemental Material [file TVEQ_A_2142318_SM2522.docx]

**Word Cloud and occurrences of keywords**

Pubmed analysis was performed using a total of 309 results. In the word cloud analysis (fig. 1) we can observe that “phytotherapy” was the most prevalent. In table 1 we can observe the exact occurrence of each of the keywords of interest in order of higher to lower occurrence.

**
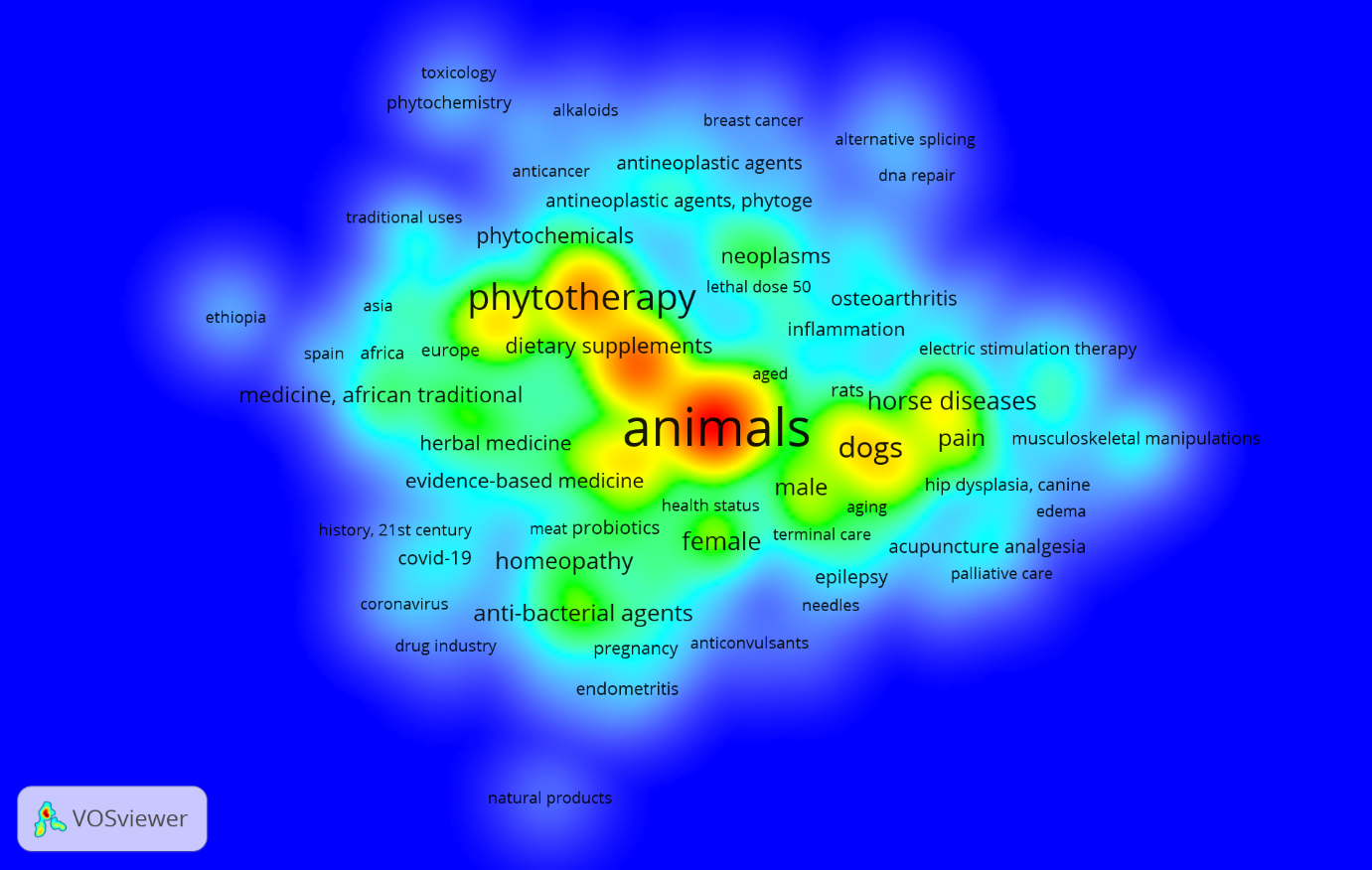
**

**Figure 1.** Resulting wordcloud of total keywords detected in the PubMed search

**Table 1.** Occurrence of keywords of interest after Pubmed search

| **Keywords** | **Occurence** |
| --- | --- |
| Phytotherapy | 110 |
| Plants, medicinal | 63 |
| Plant extracts | 51 |
| Acupuncture therapy | 46 |
| medicine, traditional | 31 |
| medicine, Chinese traditional | 25 |
| plant preparations | 23 |
| homeopathy | 22 |
| phytochemicals | 19 |
| ethnobotany | 18 |
| ethnopharmacology | 18 |
| medicine, African traditional | 16 |
| drugs, Chinese herbal | 13 |
| acupuncture points | 11 |
| herbal medicine | 11 |
| medicinal plants | 10 |
| acupuncture analgesia | 9 |
| massage | 8 |
| acupuncture | 7 |
| physical therapy modalities | 7 |
| electric stimulation therapy | 5 |
| plant oils | 5 |
| traditional medicine | 5 |
| herbs | 4 |
| medicine, ayurvedic | 4 |
| meridians | 4 |
| phytochemistry | 4 |
| *traditional medicine | 3 |
| electroacupuncture | 3 |
| hydrotherapy | 3 |
| manipulation, chiropractic | 3 |
| massage therapy | 3 |
| chiropractic | 2 |
| herbal | 2 |
| herbal remedies | 2 |
| low level light therapy | 2 |
| medicine, east Asian traditional | 2 |
| natural products | 2 |
| physical therapy | 2 |
| phytochemical | 2 |
| traditional Chinese medicine | 2 |
| veterinary homeopathy | 2 |

Scopus analysis was performed using a total of 46 results. In the word cloud analysis (fig. 2) we can observe that “Acupuncture” was the most prevalent. In table 2 we can observe the exact occurrence of each of the keywords of interest in order of higher to lower occurrence.


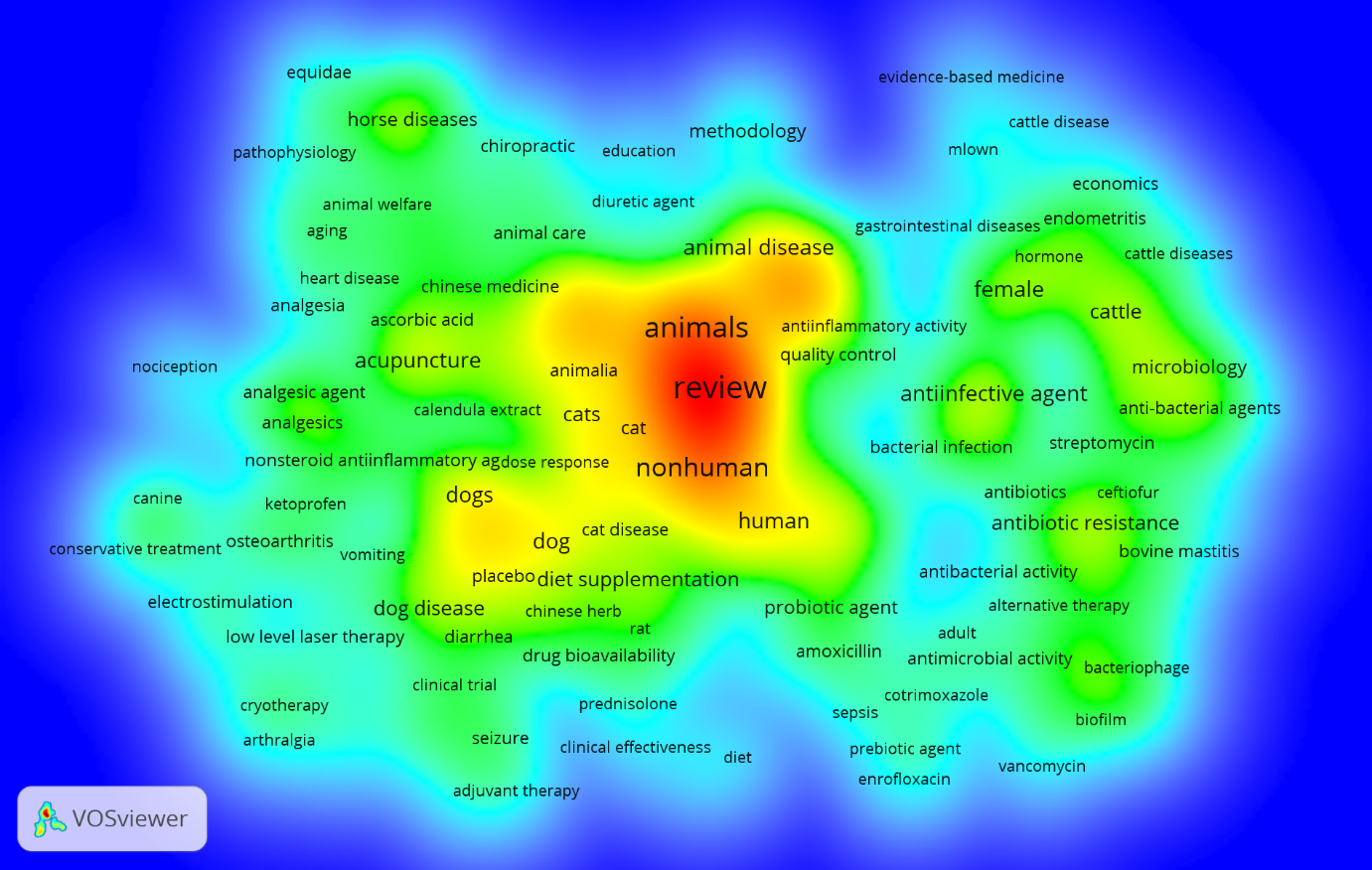


**Figure 2.** Resulting wordcloud of total keywords detected in Scopus search

**Table 2.** Occurrence of keywords of interest in Scopus search

| **Keyword** | **Occurrence** |
| --- | --- |
| acupuncture | 10 |
| herbal medicine | 6 |
| herbaceous agent | 5 |
| homeopathy | 5 |
| Chinese medicine | 4 |
| chiropractic | 4 |
| electroacupuncture | 3 |
| electrostimulation | 3 |
| low level laser therapy | 2 |
| medicinal plant | 3 |
| physiotherapy | 3 |
| Chinese herb | 2 |
| physical therapy modalities | 2 |
| plant extracts | 2 |
| plant medicinal products | 2 |
| traditional medicine | 2 |

Web of Science analysis was performed using a total of 711 results. The total word cloud analysis can be observed in fig. 3. In table 3 we can observe the exact occurrence of each of the keywords of interest in order of higher to lower occurrence, being “acupuncture” the most prevalent.


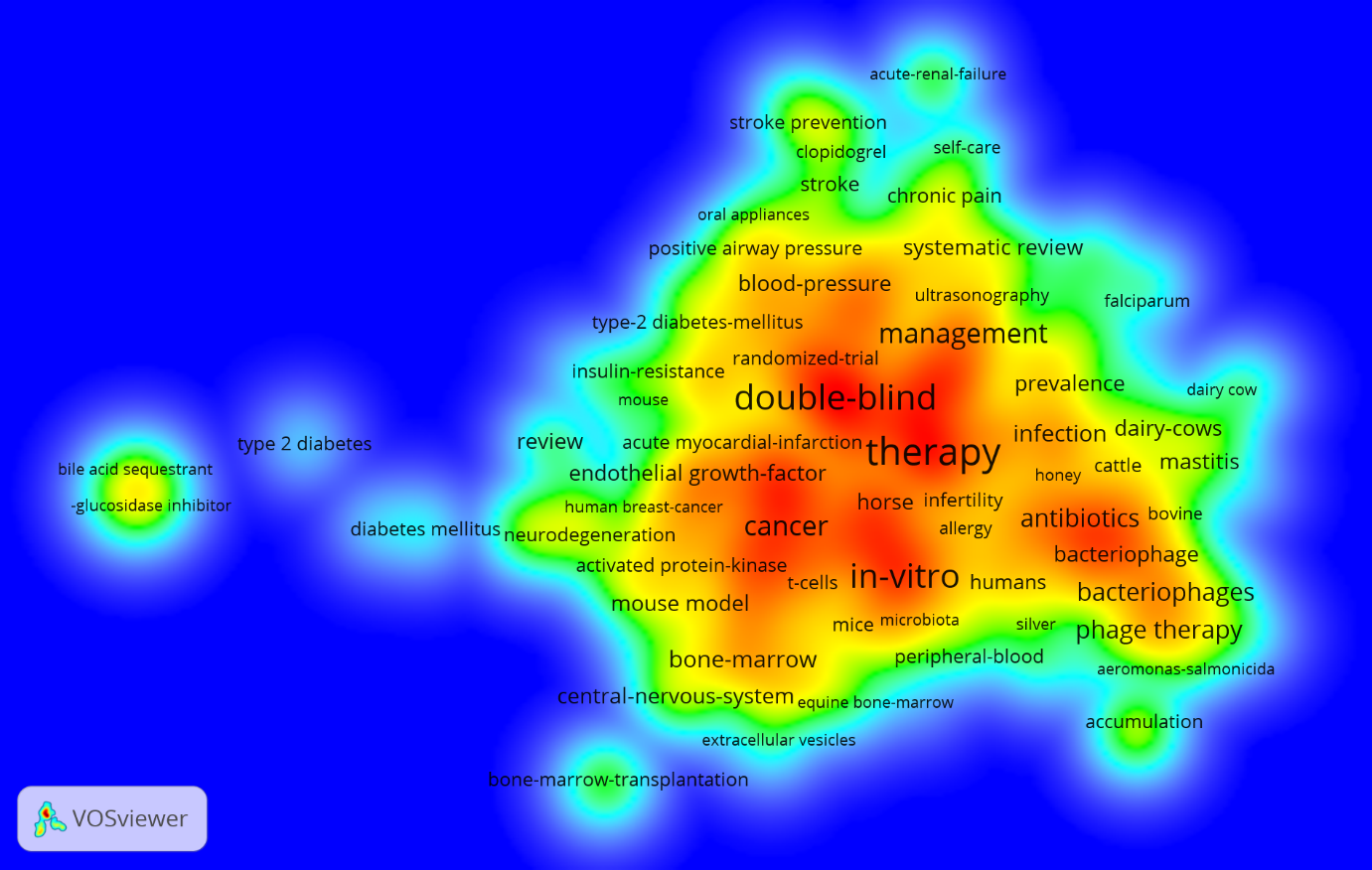


**Figure 3.** Resulting wordcloud of total keywords detected in Web of Science search

**Table 3.** Occurrence of keywords of interest in Web of Science search

| **Keyword** | **Occurence** |
| --- | --- |
| acupuncture | 8 |
| essential oils | 8 |
| phytochemicals | 8 |
| medicinal plants | 6 |
| herbs | 5 |
| natural products | 4 |
| essential oil | 3 |
| herbal medicine | 3 |
| natural products | 3 |
| phytotherapy | 3 |
| plant extracts | 3 |
| traditional Chinese medicine | 3 |
| auricular acupuncture | 2 |
| Chinese herbal medicine | 2 |
| electroacupuncture | 2 |
| homeopathy | 2 |
| physical therapy | 2 |
| plant essential oils | 2 |
| plant extracts | 2 |
